# Remote Ischemic Preconditioning Attenuates Hepatic Ischemia/Reperfusion Injury after Hemorrhagic Shock by Increasing Autophagy

**DOI:** 10.7150/ijms.51268

**Published:** 2021-01-01

**Authors:** Hao Zhou, Lin Li, Hao Sun, Hua Li, Yuxuan Wu, Xiaomin Zhang, Jinsong Zhang

**Affiliations:** Emergency Department, Nanjing Medical University First Affiliated Hospital and Jiangsu Province Hospital, NanJing City, China.

**Keywords:** Remote ischemic preconditioning, hemorrhagic shock, hepatic, ischemia-reperfusion injury, autophagy.

## Abstract

Fluid resuscitation after hemorrhagic shock is a model of systemic ischemia/reperfusion injury (SI/RI), and the liver is one of the main target organs. Ischemic preconditioning (IPC) can reduce hepatic ischemia-reperfusion injury (I/RI) via autophagy. However, whether remote ischemic preconditioning (RIPC) can alleviate the liver injury that is secondary to hemorrhagic shock and the role of autophagy in this process remain unclear. Thus, we constructed a hemorrhagic shock model in rats with or without RIPC to monitor mean arterial pressure (MAP) and investigate liver secondary injury levels via serum aminotransferase, ultrasound, HE staining and TUNEL fluorescence staining. We also detected levels of serum inflammatory factors including tumor necrosis factor-alpha (TNF-α) and interleukin 1β (IL-1β) by enzyme-linked immunosorbent assay (ELLSA), observed autophagosomes by Transmission electron microscopy (TEM), and analyzed LC3, Beclin-1, p62 protein expression levels by immunohistochemical (IHC) and western blot (WB). We found that RIPC increased blood pressure adaptability, decreased lactate (Lac) and aminotransferase levels, and delayed the decrease in liver density. Levels of inflammatory factors TNF-α, IL-1β and apoptosis were attenuated, autophagosomes was increased in the RIPC group compared with controls. IHC and WB both revealed increased LC3 and Beclin-1 but decreased p62 protein expression levels in the RIPC group. Together, our data suggest that RIPC-activated autophagy could play a protective role against secondary liver injury following hemorrhagic shock.

## Introduction

Hemorrhagic shock after severe trauma and the subsequent massive fluid resuscitation are a type of SI/RI that often results in the release of inflammatory factors and remote organ injury 1, 2. The liver is one of the main target organs of SI/RI, and previous studies have shown that hepatic I/RI is related to mitochondrial dysfunction, excessive oxygen free radicals, and increased apoptosis 3, 4.

Autophagy is the cellular process in which damaged organelles and macromolecules are degraded by cytoplasmic lysosomes and is regulated by many highly conserved autophagy-related genes. Autophagy eliminates damaged organelles, increases ATP and amino acid levels to stabilize cell morphology, maintain function, or promote apoptosis 5-7. Hepatocytes have an abundance of mitochondria and autophagy plays a crucial role in hepatic I/RI 8-10.

IPC is the process of pre-programmed transient ischemia-reperfusion, which promotes tissue adaptation to prolong I/RI. IPC can effectively alleviate liver transplantation-induced I/RI, which has been confirmed by many animal experiments and clinical data, but the specific molecular mechanism remains unclear 3, 11-13. Degli et al. suggested that IPC might protect the transplanted liver by activating autophagy and attenuating apoptosis 11. Previous animal studies have also confirmed that IPC combined with rapamycin can attenuate hepatic I/RI in aged rats 13.

However, hepatic IPC can only be performed preoperatively, and is not applicable for other acute liver injuries. Studies have shown that RIPC can reduce I/RI of transplanted liver via autophagy 3, 14. However, whether RIPC can attenuate secondary liver injury caused by systemic injury, such as hemorrhagic shock, remains unclear. Thus, we constructed a rat model of hemorrhagic shock to investigate the protective mechanism of RIPC and the role of autophagy.

## Materials and Methods

### Animal preparation

This study was approved by the Animal Ethical and Welfare Committee of Nanjing Medical University (IACUC-1811045), and was conducted in accordance with the Guide for the Care and Use of Laboratory Animals (NIH publication No. 85-23, revised 1996). Eighty adult male Sprague-Dawley rats (Vital River Biological Co., Ltd., Beijing, China), weighing 350±20 g, were kept in a temperature (22°C-26°C) and humidity (45%-75%) controlled room on a 12 h light-dark cycle with free access to standard chow and tap water in a specific-pathogen-free animal facility. There was no significant difference in body weight between the two groups. The animals were fasted overnight except for free access to water and were anesthetized with urethane (800 mg/kg, i.p.) and α-chloralose (40 mg/kg, i.p.). Additional doses of 10% of the initial dose were administered as required to maintain anesthesia. The trachea was orally intubated, and breathing was kept spontaneous. Polyethylene catheters were cannulated into the right carotid artery for continuous MAP monitoring (ADInstruments, Sydney, Australia), as well as for blood withdrawal and blood reinfusion. All catheters were flushed intermittently with saline containing 2.5 IU/mL of crystalline bovine heparin 15.

### Experimental procedures

After catheterization, the animals were randomized into two groups: RIPC and control. The reperfusion times were 0, 2, 6, 12, and 24 h, with n=5 rats in each group. Control (0 h) were sham treated, i.e., the rats were only subjected to catheterization, not hemorrhagic shock and resuscitation. The RIPC 0 h group were sham plus the RIPC procedure. All other rats were subjected to the same hemorrhagic shock and resuscitation procedures. Hemorrhagic shock was accomplished by a blood withdrawal of 22.5 μL per 1 g body weight at a constant speed; the blood was saved with anticoagulation. After a hypotensive period of 60 min, the animals were resuscitated with the withdrawn blood and 0.9% of normal saline equivalent to the volume of blood withdrawn. Before the initiation of hemorrhagic shock, three cycles of left femoral artery occlusion were performed using hemostatic clips for 5 min, followed by reperfusion for 5 min; this was the means of inducing RIPC. Liver density was first measured by high-resolution ultrasound (Vevo 3100, VisualSonics, FUJIFILM, Toronto, Canada) in living animals at the indicated timepoint after reperfusion. Then 3 mL of blood was collected, the animals were sacrificed, and liver tissues were harvested.

### Serum examination

Serum levels of TNF-α, IL-1β, and Lac were quantitated from blood samples using commercially available ELLSA kits according to the manufacturer's instructions (HuiJia Biotechnology, Xiamen, China). ALT and AST levels (markers of hepatocellular injury) were measured in blood samples by the Animal Diagnostic Laboratory (Servicebio Technology Co., WuHan, China).

### TEM

The hepatic sections were prepared for TEM to investigate ultrastructural changes following SI/RI and RIPC. Briefly, rats under anesthesia were perfused from the left ventricle of the heart with normal saline and then with 2% paraformaldehyde plus 2% glutaraldehyde in 0.1 M phosphate buffer (pH 7.4). The liver was removed and cut into 1-mm3 blocks that were fixed in 2.5% glutaraldehyde overnight at 4°C. After washing with PBS (pH 7.4), the tissue blocks were fixed in 1% osmic acid, gradually dehydrated with ethanol and acetone, embedded in epoxy resin, and incubated at 70°C for 48 h to allow for resin polymerization. Ultrathin sections were cut, double stained with uranyl acetate and lead citrate, and then examined under a Hitachi H-600 TEM (Hitachi, Tokyo, Japan).

### IHC examination

The right lobe of the liver was sagittally sliced into 1.5-cm slices, which were then fixed in 10% formalin for 24 h at room temperature (20°C-25°C), dehydrated through a graded ethanol series, cleared in xylene, embedded in paraffin wax, and cut into 5-µm sections. Histological observations were performed after hematoxylin and eosin (HE) staining; apoptosis was observed using TUNEL fluorescence staining (Yeasen, ShangHai, China). IHC staining was performed using the following primary antibodies: anti-LC3 (1:200; Cell Signaling Technologies, Danvers, MA, USA), anti-Beclin-1 (1:300; Abcam, Cambridge, UK), and anti-p62 (1:200; Abcam). Following incubations with primary and secondary antibodies, SABC and DAB color development were performed according to the manufacturer's protocols. Images of sections were captured using a light microscope; four fields of each section were randomly selected, and the positive expression rate and gray values of stained areas were measured under the same light intensity to semi-quantify protein expression levels using Image-Pro Plus 6.0 (Media Cybernetics, Inc., Rockville, MD, USA).

### Western blot analysis

Hepatic tissue sections were lysed in RIPA lysis buffer, and then total protein was extracted, and protein concentrations were measured using the Bradford assay. Protein samples (10 µg) were separated by 12% SDS-PAGE and transferred to polyvinylidene fluoride (PVDF) membranes (EMD Millipore, Billerica, MA, USA). The membranes were then incubated with the same primary antibodies used for IHC (anti-LC3, 1:1000; anti-Beclin-1, 1:1200; and anti-p62, 1:1000) overnight at 4°C, followed by incubation with horseradish peroxidase-conjugated secondary antibody for 2 h at 37°C. An enhanced chemiluminescence kit (Beyotime Institute of Biotechnology, Haimen, China) was used to visualize immunoreactive bands, and the results were analyzed using Quantity One software (version 4.62; Bio-Rad Laboratories Inc., Hercules, CA, USA).

### Statistical analysis

All experimental data are presented as mean ± standard deviation, and were analyzed using SPSS 24.0 software (IBM Corp., Armonk, NY, USA). All experiments were independently repeated five times. All data were analyzed by shapiro-wilk test and conform to a normal distribution. Differences among groups were analyzed by the independent-samples t-test or single-factor analysis of variance. IHC data were analyzed by ANOVA followed by Bonferroni's post-hoc test. P<0.05 was considered to indicate a statistically significant difference.

## Results

### Increased adaptive MAP in the RIPC group during the ischemic phase

**Figure 1:**
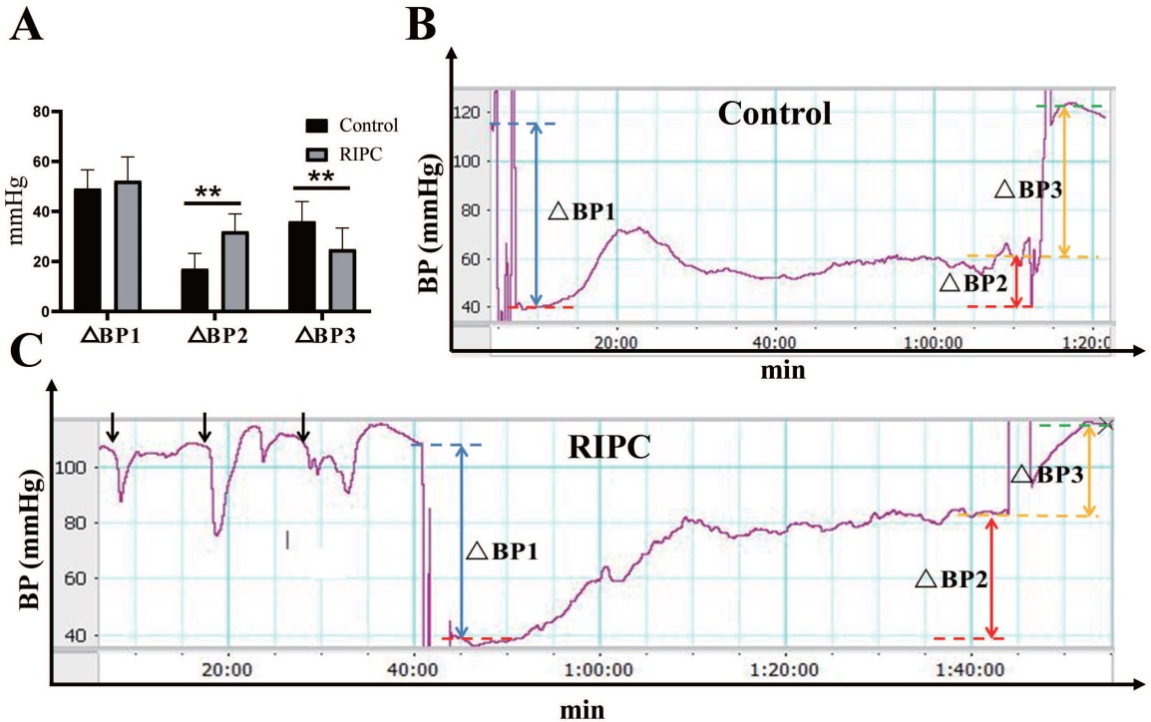
** A.** Blood pressure (BP) analysis. **B.** Trends of BP change in the control. **C.** Trends of BP change in the RIPC. Blue dotted line indicates basal MAP value, red dotted line indicates MAP value in the early phase of ischemia, yellow dotted line indicates MAP value in the late phase, and green dotted line indicates MAP value after resuscitation. ΔBP1 indicates the fall in blood pressure after equal amount of ischemia, indicated by blue double arrows. ΔBP2 indicates blood pressure in the late phase of ischemia minus that in the early phase, which represents the degree of blood pressure adaptation in the ischemic phase, indicated by red double arrows. ΔBP3 indicates the rise in magnitude of blood pressure after resuscitation, indicated by yellow double arrows. Black arrows indicate changes in blood pressure during RIPC. **P < 0.01; *n*=20 rats in each group.

During RIPC, the arterial blood pressure showed a rapid decrease in MAP immediately after clamping the left femoral artery, with a decrease of 16±5 mmHg. Then, MAP gradually increased and stabilized to a basal value after relaxation. There was no statistical difference in the MAP decline between the two groups after equal amounts of ischemia. During the ischemic phase, the MAP in rats increased adaptively even without fluid resuscitation. The increase in MAP in the RIPC group was significantly higher than that in the control group. Therefore, in the late phase of ischemia, MAP was significant higher in the RIPC group than that in the control group. After resuscitation, the increase of MAP in the RIPC group was significantly lower than that in the control group (Fig. 1). The mortality of control group and RIPC group were 28.13% (9/32) and 11.43% (4/32) respectively.

### Reduction of Lac, inflammatory factors, and markers of liver injury after RIPC

**Figure 2:**
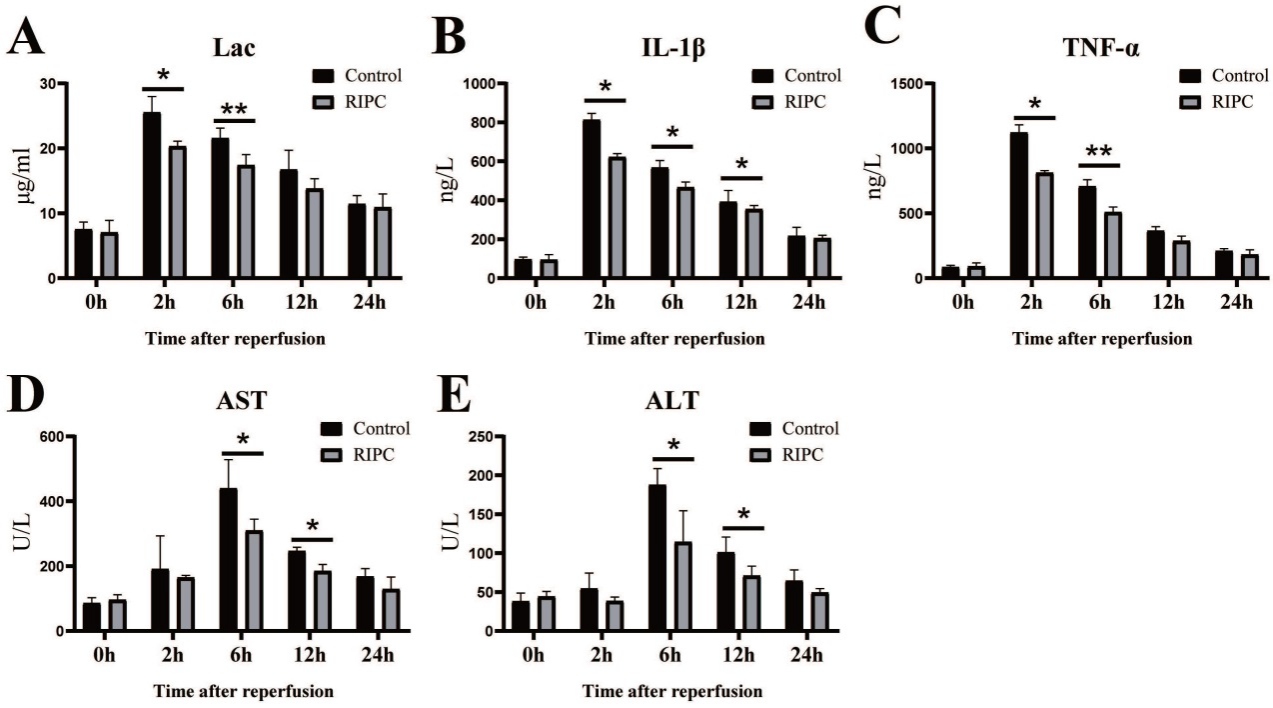
The changes of Lac, inflammatory factors (IL-1β, TNFα), and markers of liver injury (ALT, AST) after SI/RI. *P < 0.05; **P < 0.01; n=5 rats in each timepoint.

Lac is a good index of shock severity 18. We found that Lac peaked at 2 h after SI/RI, and then gradually declined. RIPC significantly reduced Lac at 2 and 6 h (Fig. 2A). SI/RI led to a significantly increase in the pro-inflammatory factors IL-1β and TNFα, which also peaked at 2 h. Levels of pro-inflammatory factors in the RIPC group were significant lower than those in the control group (Fig. 2B/C). Liver injury marker (ALT, AST) peaks occurred later than Lac and inflammatory factor peaks, appearing 6 h after SI/RI. ALT and AST were also significant higher in the control group than those in the RIPC group at 6 and 12 h (Fig. 2D/E).

### RIPC reduced apoptosis and delayed the decrease in liver density

**Figure 3:**
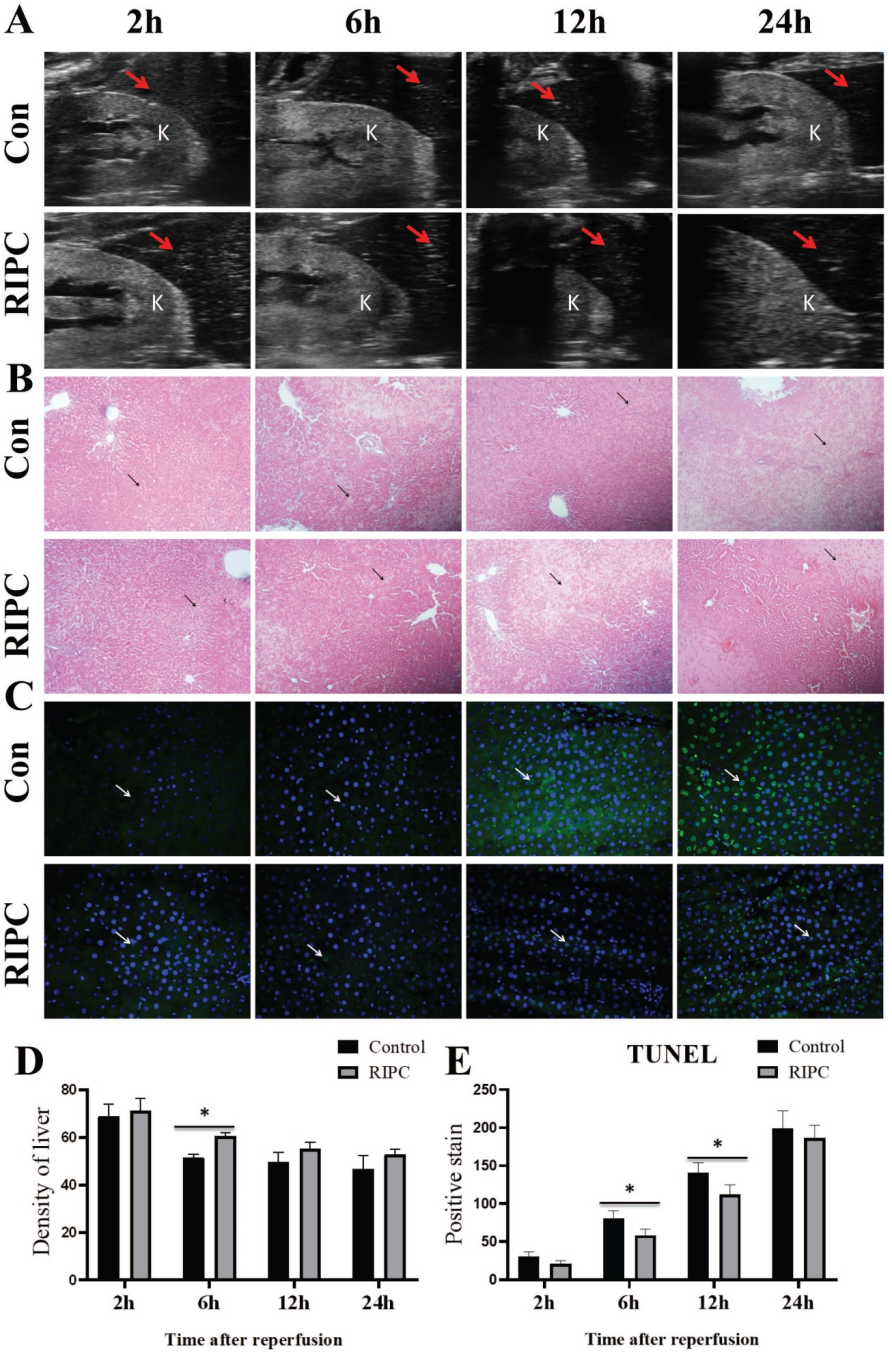
** A.** Liver and kidney plane ultrasound image, where the red arrow indicates the density detection area in the liver, and K indicates the kidney. **B.** The histological changes are observed by HE-stained, arrow indicates the area of ballooning changes in the liver; magnification: 100×. **C.** TUNEL fluorescence staining showing that hepatocellular apoptosis after SI/RI, arrow indicates positive TUNEL fluorescence staining; magnification: 400×. A-C in the same row are images from samples take 2, 6, 12, and 24 h after I/RI (left to right). *P < 0.05; n=5 rats in each timepoint.

Liver ultrasound showed that the liver density decreased over time after SI/RI, also presenting with heterogeneous increases. The liver density in the RIPC group was higher than that in the control group at 6 h (Fig. 3A/D). As shown in Fig. 3B, at 2 h after SI/RI, HE staining showed swelling and ballooning degeneration in hepatocytes. At 6 h, the ballooning degeneration agglomerated to form a film, with inflammatory infiltration in the local portal area, and the hepatic sinus structure had disappeared. These changes were significantly alleviated in the RIPC group. At 12 and 24 h, a large area of ballooning hepatocytes was observed, and the destruction of hepatic sinus structure was further increased, presenting with a large number of pyknotic and hematoxylin-stained nuclei and the infiltration of multinucleated cells. In the RIPC group, the ballooning area was smaller, with more normal sinusoidal structures. TUNEL staining illustrated that hepatocellular apoptosis was gradually increased with prolonged SI/RI time. Apoptotic cells were significantly less frequent in the RIPC group than that in the control group at 6 and 12 h (Fig. 3C/E).

### Increased autophagy in the RIPC group

**Figure 4:**
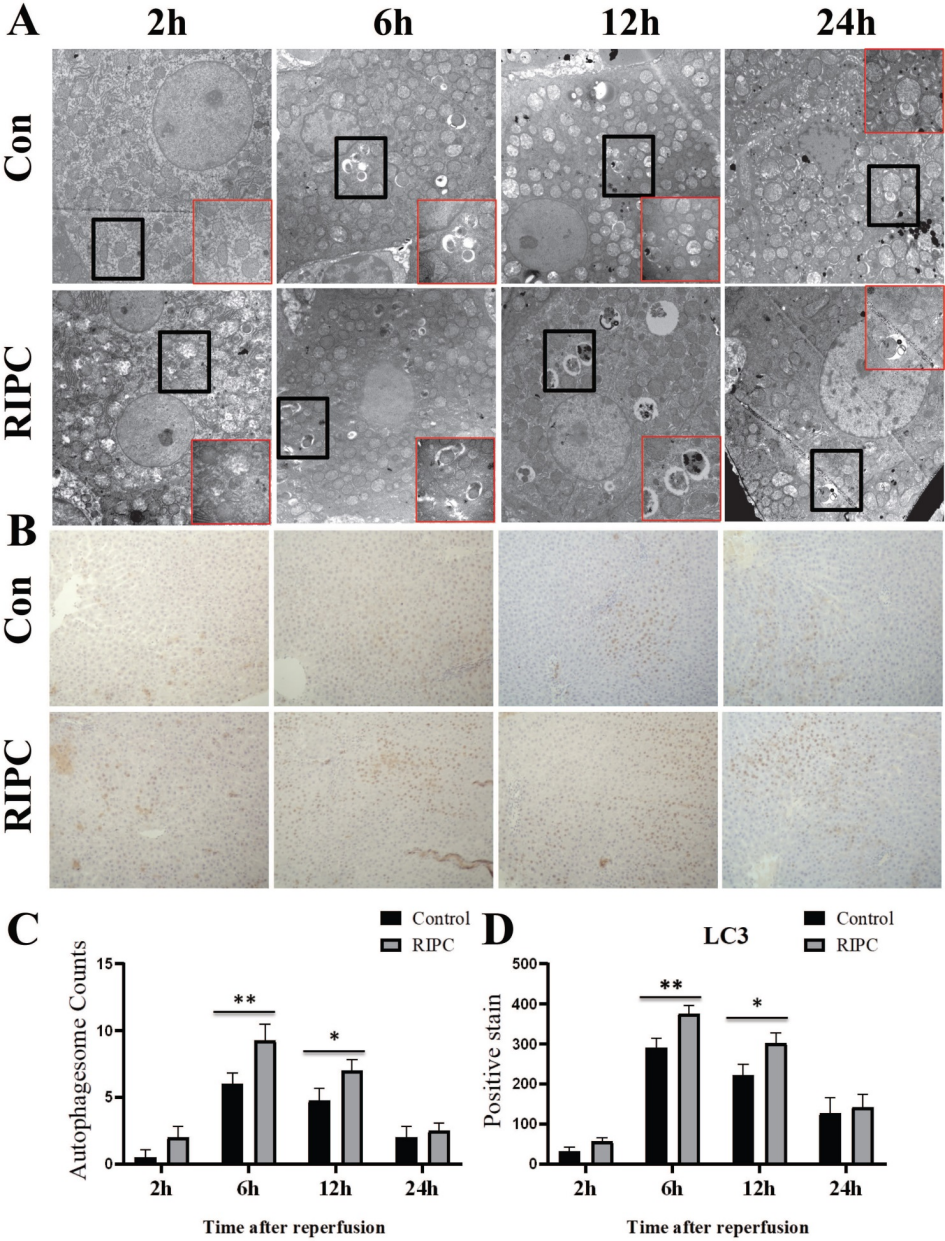
TEM reveal ultrastructural changes after SI/RI; magnification: 8000×; local magnification: 30000×. **B.** LC3 IHC after SI/RI magnification: 200×. A-B in the same row are images from samples take 2, 6, 12, and 24 h after I/RI (left to right). *P < 0.05; **P < 0.01; n=5 rats in each timepoint.

The obvious swelling of mitochondria in hepatocytes was observed by TEM at 2 h after SI/RI. Many autophagosomes were observed in the hepatocytes at 6 h after SI/RI, and the number per single cell in the RIPC group was more than that in the control group. At 12 and 24 h after SI/RI, the number of autophagosomes in the control group had gradually decreased, and disintegrated subcellular organelles could be observed, as well as nuclear condensation at 24 h. There were still more autophagosomes in the RIPC group at these timepoints, with the subcellular organelle structure wrapped by a double membrane. The degree of nuclear condensation was also lower than in the control group (Fig. 4A/C). IHC showed that LC3 was marginally expressed at 2 h after SI/RI, peaked at 6 h, and then gradually decreased at 12 and 24 h. LC3 expression was significant higher in the RIPC group than that in the control group at 6 and 12 h (Fig. 4 B/D).

### Higher Beclin-1 and lower p62 expression in the RIPC group

**Figure 5:**
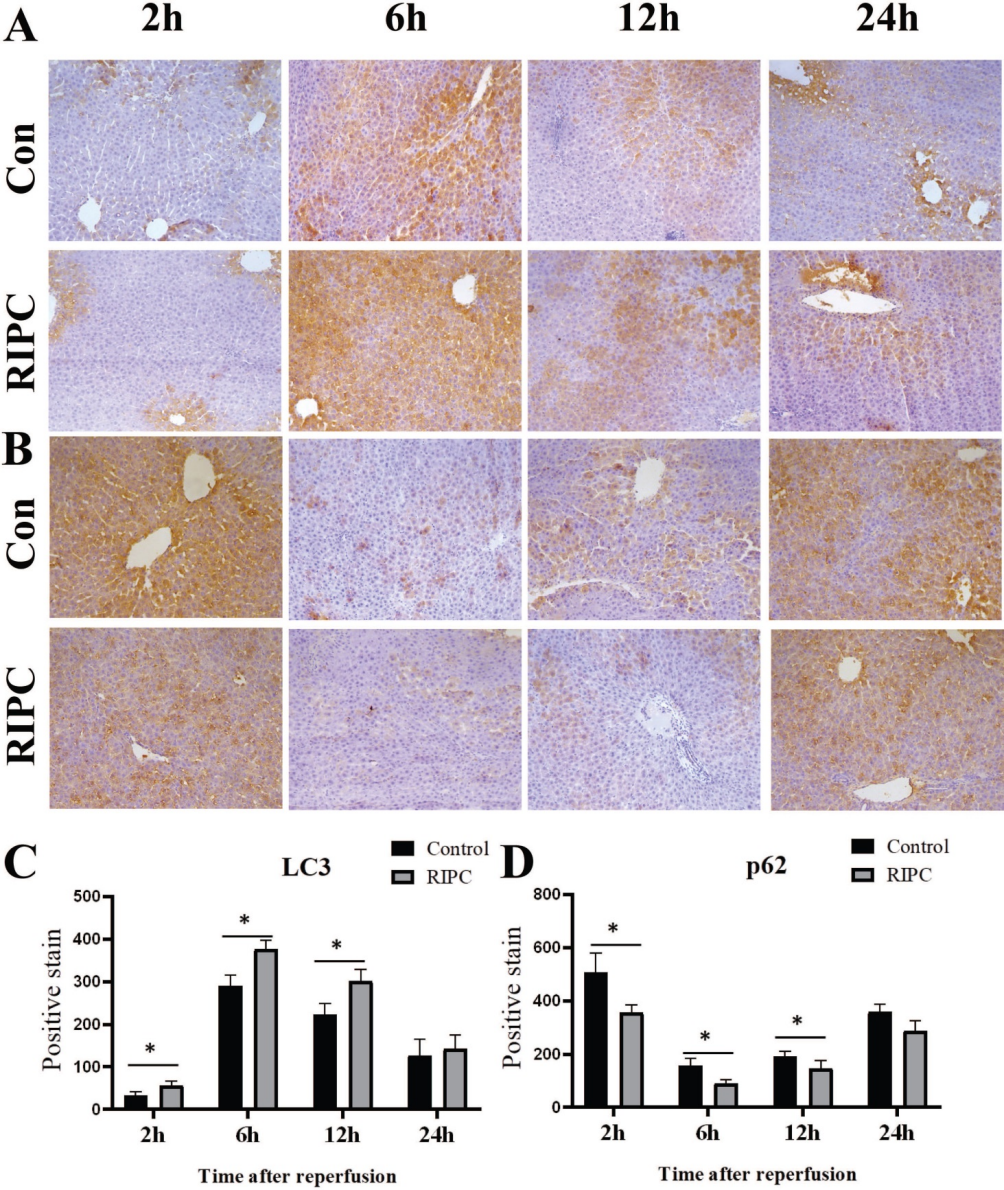
** A.** Beclin-1 IHC after SI/RI, magnification: 200×. **B.** p62 IHC after SI/RI, magnification: 200×. A-B in the same row are images from samples take 2, 6, 12, and 24 h after I/RI (left to right). *P < 0.05; n=5 rats in each timepoint.

Beclin-1 IHC indicated that its expression trend was synchronized with LC3, and there was a slight expression at 2 h after SI/RI, with the peak at 6 h, and then a gradual decrease. Beclin-1 expression was significant higher in the RIPC group than that in the control group at 2, 6, and 12 h (Fig. 5 A/C). At 2 h after SI/RI, p62 expression was observed, and the staining was decreased at 6 h, and then gradually increased after 12 and 24 h. At 2, 6 and 12 h, p62 expression was significant lower in the RIPC group than that in the control group (Fig. 5B/D).

### Semi-quantitative WB analysis of LC3, Beclin-1, and p62 expression

**Figure 6:**
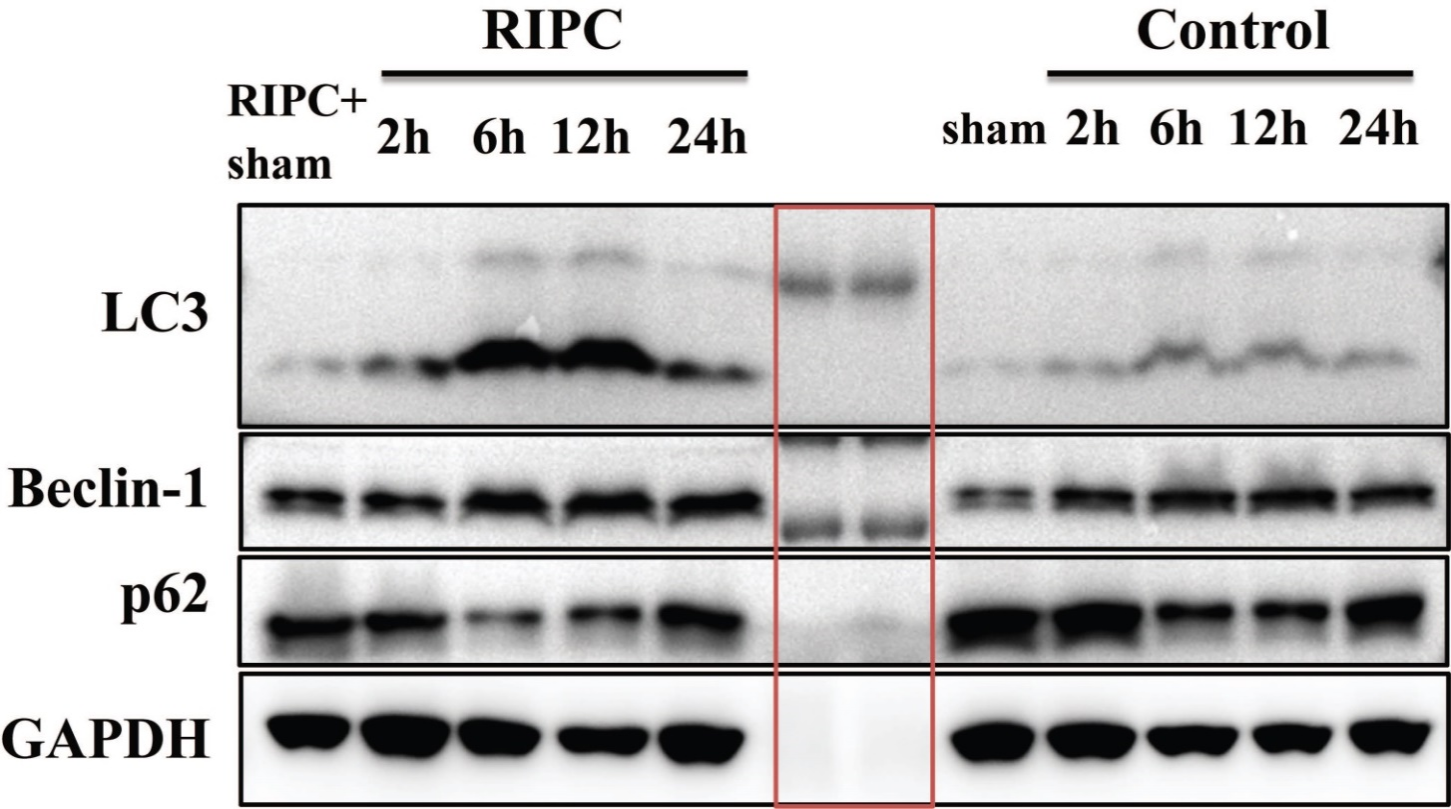
LC3, Beclin-1, and p62 levels in liver tissues as detected by semi-quantitative WB. The bands are markers in the red box. n=5 rats in each timepoint.

WB semi-quantitative detection showed that LC3 expression was significant higher in the RIPC group than that in the control group at 6 and 12 h after SI/RI. Beclin-1 expression was also significantly increased in the RIPC group compared with the control group at 6 and 12 h after SI/RI. p62 expression was lower in the RIPC group than that in the control group, and the difference was significant at 2, 6, and 12 h after SI/RI. These data showed trends that were consistent with the IHC results (Fig. 6).

## Discussion

RIPC is a method with potential application to protect important organs. It is simple, safe and effective to apply IPC to limbs or muscle groups instead of directly applying to high-risk target organs, such as the heart and liver via surgery 2, 19. However, the current RIPC procedure is controversial, without a uniform standard for the length of time and number of cycles. It has been reported that the timing of RIPC procedures can affect efficacy 3, 11, 20. Xie et al. observed that RIPC with 5 min ischemia and 5 min relaxation for 3 cycles could effectively reduce troponin I levels after cardiac surgery 21. In this study of RIPC, we also found that RIPC effectively reduced the secondary increase in ALT and AST after hemorrhagic shock, even with shorter circulation times and fewer cycles. Meanwhile, we observed that the ballooning degeneration of hepatocytes was decreased after RIPC, with more normal sinusoidal structures observed. Furthermore, liver density, as determined by ultrasound, was improved compared with the control group.

In this study, RIPC caused a blood pressure fluctuation of 16 ± 5 mmHg, but did not affect the basal MAP value. After clamping the artery, blood pressure decreased rapidly and then increased, which might be attributed to self-regulation of tissue transient ischemia. In the early phase of ischemia, there was no significant difference between the two groups in the decreased MAP caused by equal amount of ischemia, but the RIPC group had a more significant increase in self-adaptive blood pressure than the control group. After fluid resuscitation of the same volume, the increase in blood pressure in the RIPC group was also significantly lower than in the control group. It has been reported that in 50-60% blood volume ischemia, MAP was decreased to approximately 40 mmHg 17, 22. Hu X et al. also found that the RIPC group had higher MAP than the control group in the shock phase 17. Studies have proved that in hemorrhagic shock, the ischemia and reperfusion condition in microcirculation is almost the same as that in large circulation 23; thus, the increased MAP during ischemia may promote perfusion of local organs. Therefore, RIPC may increase perfusion of important organs during ischemia by improving self-adaptation to ischemia in the body, while also reducing the blood pressure fluctuation during reperfusion. Lac levels in the RIPC group were lower than in the control group, suggesting that organ perfusion in the RIPC group was better.

Activation of inflammatory factors is an important factor during I/RI 2,3. Anti-inflammatory and anti-apoptotic effects may be important mechanism for the protective functions of RIPC for organs 3,16. Systemic inflammatory response is over-activated during hemorrhagic shock, and the expression of pro-inflammatory factors, such as TNF-α are significantly increased 22,24. Inhibiting these inflammatory responses by drugs can effectively alleviate target organ injury after hemorrhagic shock 4,24. In this study, we found that expression of the pro-inflammatory factors IL-1β and TNF-α were decreased in the RIPC group compared with the control group, and there was more preservation of sinusoidal tissue structure in the RIPC group than in the control group by HE staining. Additionally, TUNEL staining showed that hepatocellular apoptosis was significantly reduced compared with the control group. Thus, we hypothesize that under hemorrhagic shock, RIPC may play a protective role in hepatocytes by the reducing release of systemic pro-inflammatory cytokines and inhibiting apoptosis.

Autophagy is a catabolic pathway triggered under various stress conditions, including ischemia and hypoxia, which aims to restore intracellular ATP and amino acids and eliminate damaged organelles. Studies have suggested that autophagy interacts with both the apoptotic and necrotic pathways 25. Degli et al. showed that IPC attenuated primary hepatic I/RI by increasing autophagy in fatty liver patients undergoing transplantation 11. Bone marrow mesenchymal stem cell-derived hepatocyte-like cell Exosomes and ulinastatin have also been proven to reduce hepatocellular apoptosis by enhancing autophagy 26, 27. Reducing mitochondrial autophagy by knocking out PINK can increase hepatic I/RI 28. Our study demonstrated that in SI/RI-induced secondary liver injury, RIPC increased the expression of autophagy-related proteins, such as LC3 and Beclin-1, and decreased hepatocellular apoptosis, as well as markers of liver injury. Therefore, we hypothesize that RIPC-activated autophagy plays a protective role in hepatic I/RI secondary to hemorrhagic shock.

There are some limitations to this study. First, we only validated the protective effect of RIPC on organs with hemorrhagic shock. It has been reported that programmed transient ischemia-reperfusion during shock, or during resuscitation also has protective effects 16. Second, the protective effect of IPC is more prominent in primary hepatic I/RI in special cases, such as fatty and senile liver 10, 13, 29. This study showed that RIPC-activated autophagy plays a role in hepatoprotection after hemorrhagic shock, but to what extent autophagy is the underlying mechanism and whether RIPC plays a more prominent role in special groups need further investigation.

## References

[1] Cannon JW (2018). Hemorrhagic Shock. N Engl J Med.

[2] Vourc'h M, Roquilly A, Asehnoune K (2018). Trauma-induced Damage-Associated Molecular Patterns-Mediated Remote Organ injury and immunosuppression in the Acutely ill Patient. Front Immunol.

[3] Weili Yang, Ji Chen, Jichun Yang (2018). Novel Targets for Treating Ischemia-Reperfusion Injury in the Liver. Int J Mol Sci.

[4] Chen G, Song X, Zhou H (2017). Carboxyfullerene nanoparticles alleviate acute hepatic injury in severe hemorrhagic shock. Biomaterials.

[5] Czaja MJ, Ding WX, Yin XM (2014). Functions of autophagy in normal and diseased liver. Autophagy.

[6] Scherz-Shouval R, Shvets E, Elazar Z (2007). Reactive oxygen speciesare essential for autophagy and specifically regulate the activity of Atg4. EMBO J.

[7] Klionsky DJ, Abdelmohsen K, Zorzano A, Zughaier SM (2016). Guidelines for the use and interpretation of assays for monitoring autophagy (3rd edition). Autophagy.

[8] Go KL, Lee S, Kim JS (2015). Mitochondrial dysfunction and autophagy in hepatic ischemia/reperfusion injury. Biomed Res Int.

[9] Cursio R, Colosetti P, Gugenheim J (2015). Autophagy and Liver Ischemia-Reperfusion Injury. BioMed Res Int.

[10] Zeng X, Wang S, Ye Q (2019). Hypothermic oxygenated machine perfusion alleviates liver injury in donation after circulatory death through activating autophagy in mice. Artif Organs.

[11] Degli Esposti D, Sebagh M, Lemoine A (2011). Ischemic preconditioning induces autophagy and limits necrosis in human recipients of fatty liver grafts, decreasing the incidence of rejection episodes. Cell Death and Dis.

[12] Zhang Y, Shen Q, Zheng S (2018). Hepatic Ischemic Preconditioning Alleviates Ischemia-Reperfusion Injury by Decreasing TIM4 Expression. Int J Biol Sci.

[13] Jiang T, Zhan F, Wang X (2019). Combined ischemic and rapamycin preconditioning alleviated liver ischemia and reperfusion injury by restoring autophagy in aged mice. Int Immunopharmacol.

[14] Ruan W, Liu Q, Xu J (2016). Limb remote ischemic preconditioning attenuates liver ischemia reperfusion injury by activating autophagy via modulating PPAR- gamma pathway. Zhong Nan Da Xue Xue Bao Yi Xue Ban.

[15] Shen YH, Chen XR, Li P (2018). Alamandine injected into the paraventricular nucleus increases blood pressure and sympathetic activation in spontaneously hypertensive rats. Peptides.

[16] Leung CH, Caldarone CA, Rotstein OD (2015). Remote Ischemic Conditioning Prevents Lung and Liver Injury After Hemorrhagic Shock/Resuscitation: Potential Role of a Humoral Plasma Factor. Ann Surg.

[17] Hu X, Yang Z, Tang W (2014). Remote ischemic preconditioning mitigates myocardial and neurological dysfunction via K(ATP) channel activation in a rat model of hemorrhagic shock. Shock.

[18] Reynolds PS, Song KS, Wayne Barbee R (2015). Hypertension and vulnerability to hemorrhagic shock in a rat model. Shock.

[19] Koh WU, Kim J, Song JG (2019). Remote Ischemic Preconditioning and Diazoxide Protect from Hepatic Ischemic Reperfusion Injury by Inhibiting HMGB1-Induced TLR4/MyD88/NF-κB Signaling. Int J Mol Sci.

[20] Lin J, Huang H, Zeng Z (2019). Protective Effects of Ischemic Preconditioning Protocols on Ischemia-Reperfusion Injury in Rat Liver. J Invest Surg.

[21] Xie JJ, Liao XL, Ou JS (2012). Remote ischaemic preconditioning reduces myocardial injury in patients undergoing heart valve surgery: randomised controlled trial. Heart.

[22] Warren M, Subramani K, Raju R (2017). Mitochondrial dysfunction in rat splenocytes following hemorrhagic shock. Biochim Biophys Acta Mol Basis Dis.

[23] Van Iterson M, Bezemer R, Ince C (2012). Microcirculation follows macrocirculation in heart and gut in the acute phase of hemorrhagic shock and isovolemic autologous whole blood resuscitation in pigs. Transfusion.

[24] Atal SS, Atal S (2015). Ulinastatin - a newer potential therapeutic option for multiple organ dysfunction syndrome. J Basic Clin Physiol Pharmacol.

[25] Maiuri MC, Zalckvar E, Kroemer G (2007). Self-eating and self-killing: crosstalk between autophagy and apoptosis. Nat Rev Mol Cell Biol.

[26] Yang B, Duan W, Chen Z (2020). Bone Marrow Mesenchymal Stem Cell-Derived Hepatocyte-Like Cell Exosomes Reduce Hepatic Ischemia/Reperfusion Injury by Enhancing Autophagy. Stem Cells Dev.

[27] Zhao Y, Cai H, Liang X (2019). Protective effect of ulinastatin on hepatic ischemia-reperfusion injury through autophagy activation in Chang liver cells. J Cell Biochem.

[28] Ning XJ, Yan X, Ye QF (2018). Parkin deficiency elevates hepatic ischemia/reperfusion injury accompanying decreased mitochondrial autophagy, increased apoptosis, impaired DNA damage repair and altered cell cycle distribution. Mol Med Rep.

[29] Sikalias N, Karatzas T, Kouraklis G (2018). Intermittent Ischemic Preconditioning Protects Against Hepatic Ischemia-Reperfusion Injury and Extensive Hepatectomy in Steatotic Rat Liver. J Invest Surg.

